# Predictive role of Trimprob associated with multiparametric MRI in the diagnosis of prostate cancer

**DOI:** 10.1590/S1677-5538.IBJU.2015.0714

**Published:** 2017

**Authors:** Gustavo Cardoso Guimaraes, Walter Henriques da Costa, Renato Almeida Rosa, Stênio Zequi, Ricardo Favaretto

**Affiliations:** 1 Núcleo de Urologia, Departamento de Cirurgia Pélvica, AC Camargo Cancer Center, SP, Brasil

**Keywords:** Magnetic Resonance Imaging, Prostatic Neoplasms, Diagnosis

## Abstract

**Objectives:**

To evaluate the predictive value of TRIMprob test to detect prostate cancer (PCa) in patients referred to prostate biopsy (PB).

**Material and Methods:**

Patients with PSA <10ng/mL and rectal exam without findings suggestive of prostate cancer were selected for TRIMprob evaluation. Exam was performed by a single operator through transperineal approach. Patients admitted for the study were submitted to TRIMprob and multiparametric magnetic resonance (mpMRI) and posteriorly to PB.

**Results:**

In total, 77 patients were included. TRIMprob showed evidences of PCa in 25 (32.5%) and was negative in 52 patients (67.5%). The rate of detection of prostate cancer at biopsy was higher in patients with positive TRIMprob (16/25; 64.0%) than in patients with negative TRIMprob (11/52; 21.1%; p<0.001). Sensitivity, specificity, positive predictive value, negative predictive value and accuracy of TRIMprob were respectively 61.5%, 82.0%, 64.0%, 80.3% and 74.0%. ROC curve showed the following areas under the curve values for TRIMprob, mpMRI and combination of TRIMprob + mpMRI: 0.706; 0.662 and 0.741 respectively. At combined analysis, when both TRIMprob and mpMRI were negative for prostate cancer, accuracy was 96.3% or only 1 in 27 PB was positive (3.7%).

**Conclusions:**

Trimprob had similar predictive value for PCa in patients submitted to PB as mpMRI. Combined TRIMprob and mpMRI showed higher accuracy than when performed singly.

## INTRODUCTION

Prostate cancer (PCa) is the most common malign tumor and the second cause of death due to tumor in men worldwide (1). North American data estimate that one in every six men will present PCa, while one in every 36 men will die due to that disease. Since it affects mainly men between 50 and 70 years, it is an important health issue (2). Since population older than 60 years old will triple in the World and reach 2 billion people around 2050, it is expected a natural increase of PCa incidence (3).

The impact of population screening of Pca based on rectal exam and PSA has been continuously debated. It is been discussed the real benefit to detect a high number of patients with clinically insignificant disease and the impact on quality of life due to treatment (4). In general, PSA elevation is followed by prostate biopsy (PB). This procedure has complication risks such as hematuria (22.0%) and hemospermia (50.0%). Fever is relatively rare (3.5%) as well as sepsis with the need of hospitalization (0.5% of all biopsied men) (5).

Although with well-established indication criteria, around 75% of PB do not show malignancy, with high psychological stress of the patients (6).

In order to reduce the number of unnecessary PB and their associated morbidity, some groups suggest the inclusion of multiparametric magnetic resonance of prostate (mpMRI) for clinical decision (7, 8). mpMRI shows high accuracy for clinically significant PCa detection confirmed during radical prostatectomy (7). mpMRI detects more than 90% of clinically significant prostate tumors. However, it is less reliable to detect small tumors (<0.5mL), low grade disease (Gleason score 6) or tumors at the transition zone (8). However, its high cost avoids the use of mpMRI for population screening in our country.

In 1992, Clarbruno Vedruccio, an Italian physicist, patented a maser (“microwave amplification by stimulated emission of radiation”), a device that produces electromagnetic waves to detect anomalies of biologic tissues. The equipment TRIprob ((TRIMprobe; Finmeccania, Rome, Italy) includes a non-linear oscillator in a cylindrical probe, an analyzer of radiofrequency spectrum and a computer software (9). The probe emits electromagnetic radiation with three frequencies: 465, 930 and 1395MHz. The spectrum analyzer, powered by a receiving antenna, measures signal intensity that are visualized in a computer screen, in three different colors: red, green and blue. The interaction of the electromagnetic field emitted by the probe and the cancerous tissue results in a significant reduction of signal intensity in 465MHz (red bar), while the signals at 930 (green) and at 1395 (blue) do not change. The objective of our study was to evaluate the utility of TRIMprob test as possible screening method to identify patients candidate to transrectal prostate biopsy (9, 10).

## MATERIAL AND METHODS

This is cross-sectional study that included 398 consecutive patients submitted to TRIMprob evaluation from 2012 to 2015 in our institution. All patients with suspicion of prostate cancer and candidate to PB were submitted to TRIMprob. Criteria for PB were determined by the physician and were based on PSA level alteration such as: PSA higher than 2.5ng/mL in patients up to 55 years old and above 4.0ng/mL for patients over 55 years; free PSA/total PSA ratio lower than 20%; velocity of increase of PSA superior to 0.75ng/mL/year and mpMRI of prostate with suspicion of tumor.

**Figure 1:**
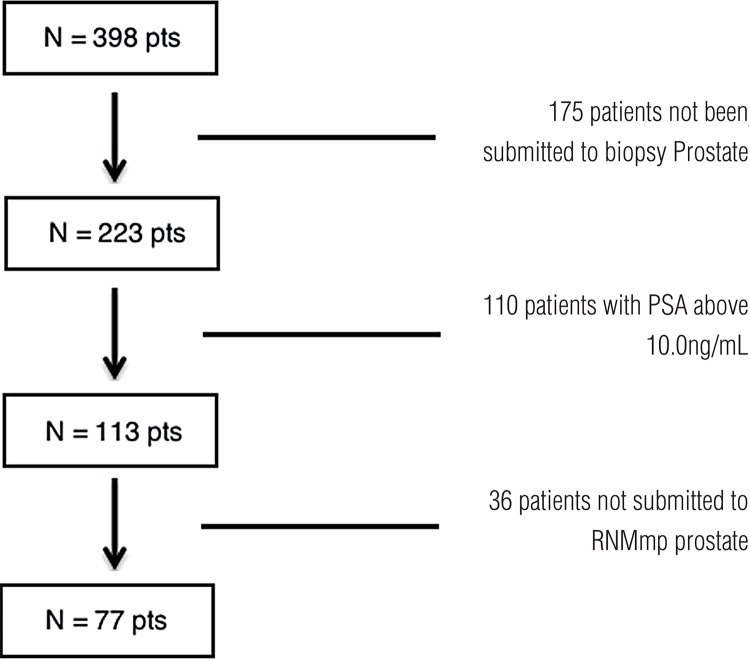
Study design–175 patients not submitted to prostate biopsy 110 patients with PSA above 10.0ng/mL 36 patients not submitted to prostate mpMRI

The following criteria for inclusion were used to evaluate TRIMprob in patients with suspicion of prostate cancer and to compare the method with prostate mpMRI: 1) patients with PSA lower than 10.0ng/mL and rectal exam without alteration; 2) patients submitted to prostate mpMRI; 3) patients submitted to confirmatory PB and posteriorly submitted to TRIMprob. The exclusion criteria included: 1) patients submitted to previous surgical treatment; 2) patients with history of use of hormonal blockers or 5-alpha-reductase inhibitors. By the end of the study, 77 patients were selected, as illustrated in Figure-1.

In our study, TRIMprob was standardized by transperineal approach by a single operator. The device is composed by a probe, a receptor and a computer screen. The probe measures 30cm in length. Electromagnetic wave penetration (EM) depends on the frequency and dielectrical proprieties of the biological tissues. 465MHz frequency penetrates 20cm (according to calculated dielectrical proprieties of striated muscle and prostate, that are quite similar) and is more adequate for the analysis of perineal region. TRIMprob exam was performed as previously described (10). In summary, patients were dressed with their underwear, standing with the legs a little apart, while the operator positioned behind the patient. According to manufacture’s instructions, accepted abnormal values corresponded to less than 40 units during digitalization in 6 standardized and conventional positions. Likewise, aside from the detected resonance values, it was valued a typical pattern of signal reduction at 465MHz below a limit of amplitude that would correspond to the presence or not of prostate cancer. TRIMprob is regularly registered at ANVISA (National Agency of Sanitary Surveillance) under the number 2824 of September, 13^th^, 2007. mpMRIs were performed in the same device using the classification PI-RADS 1.0 for positivity criteria. Patients with PI-RADS 4 and 5 were considered positive for prostate cancer. PB was performed under local anesthesia (10mL of 1% lidocaine with a needle 22G) guided by transrectal ultrasound. All patients were submitted to biopsy by a needle 16G (11). It was obtained a medium of 17 samples (Table-1). In cases when it was detected suspected lesions at mpMRI at the prostate, the corresponding area was submitted to the collection of three additional samples. Each sample was processed individually and stained by hematoxilin-eosin.

**Table 1:** Numerical characteristics of prostate biopsies of 77 patients.

	Variation	Mean	Standard Deviation
Number of collected samples	6-30	17.0	5.225
Number of positive samples	1-10	1.85	3.016

## RESULTS

The data of all 77 patients included are shown at Table-2. Mean age was 59.72 years. PSA mean value was 4.79ng/mL (2.26-9.92ng/mL), and the mean prostate volume was 45.31g (17.6-124.0g). By the end of the study, 27 (35.0%) patients were diagnosed with prostate cancer. Final Gleason score 6 (3+3) was the most frequent at PB (40.7%). PCa was associated to higher PSA levels (P=0.021) and alterations at mpMRI (P=0.029) and TRIMprob (p<0.001).

**Table 2:** Clinical and pathological characteristics of 77 patients submitted to prostate biopsy.

Variable	Total (%)	Biopsy-	Biopsy+	P value
N	77	50	27	
**Age (mean)**		59.2	60.7	0.489
40–49 years	6 (7.7)	5 (10.0)	1 (3.7)	
50–59 years	30 (39.0)	19 (38.0)	11 (40.7)	
60–69 years	31 (40.3)	18 (36.0)	13 (48.1)	
>70 years	10 (13.0)	8 (16.0)	2 (7.4)	0.464
**PSA (mean)**		4.37	5.68	0.021
0–4.0ng/mL	31 (40.3)	23 (46.0)	8 (29.6)	
4.1–10.0ng/mL	46 (59.7)	27 (54.0)	19 (70.4)	0.162
Number of samples (biopsy) (mean)	17.0	17.31	16.65	0.646
**Gleason score**				
6	-	-	11 (40.7)	
7	-	-	7 (25.9)	
8	-	-	5 (18.5)	
9	-	-	3 (11.1)	
10	-	-	1 (3.7)	
**mpMRI**				
Negative	39 (50.6)	31 (62.0)	8 (29.6)	
Positive	38 (49.4)	19 (38.0)	19 (70.4)	0.007
**TRIMprob**				
Negative	52 (67.5)	41 (82.0)	11 (40.7)	
Positive	25 (32.5)	9 (18.0)	16 (59.3)	<0.001
**mpMRI + TRIMprob**				
Negative	27 (35.1)	26 (52.0)	1 (3.7)	
Positive	50 (64.9)	24 (48.0)	26 (96.3)	<0.001

Thirty eight of the 77 patients (49.4%) showed alterations at mpMRI suggestive of prostate cancer. TRIMprob showed alterations suggestive of malign neoplasia in 25 (32.5%) patients. Detection rate of PC at biopsy was significantly higher among patients with positive TRIMprob (16/25; 64.0%) than negative TRIMprob (11/52; 21,1%; P<0,001). At combined analysis, when mpMIR and TRIMprob were negative for PCA, only 1 in 27PB was positive (3.7%) (Table-2). Assuming that the reference standard is the finding of positive biopsies for the diagnosis of prostate cancer, sensitivity, specificity positive predictive value (PPV), negative predictive value (NPV) and accuracy of TRIMprob were 61,5%, 82.0%, 64.0%, 80.3% and 74.0% respectively (Table-3).

**Table 3:** Sensitivity, specificity, PPV, NPV and accuracy of TRIMprob and mpMRI and the combination of both in 77 patients submitted to prostate biopsy.

Exam	Sensitivity	Specificity	PPV	NPV	Accuracy
TRIMprob	61.5%	82.0%	64.0%	80.3%	74.0%
mpMRI	70.3%	62.0%	50.0%	79.4%	64.9%
TRIMprob + MRI	96.2%	52.0%	52.0%	96.2%	67.5%

**Figure 2:**
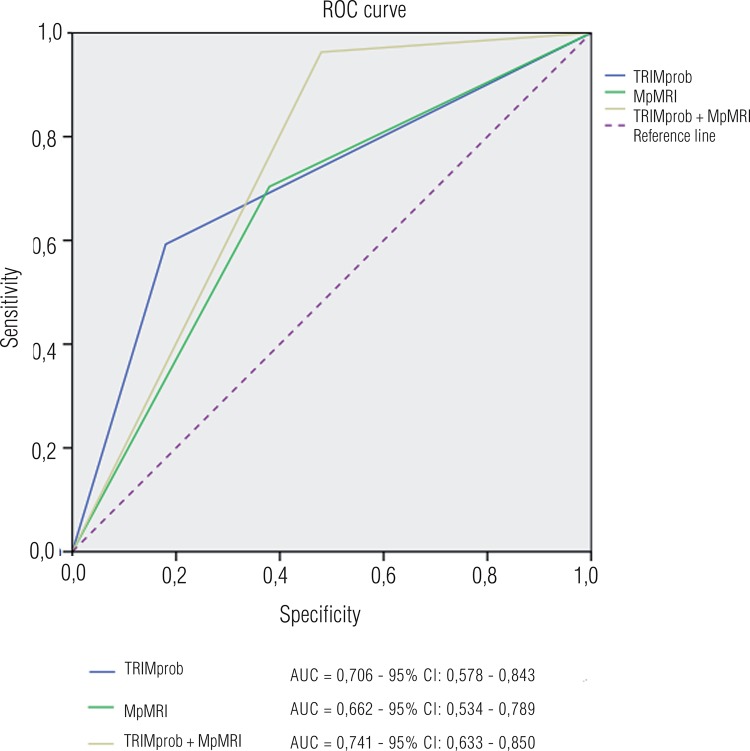
ROC curves characteristics: results of TRIMprob, mpMRI and combination of both methods in the diagnosis of prostate cancer in 77 patients submitted to prostate biopsy.

We compared the results of TRIMprob with those of mpRI. Also, we analyzed the impact of combination of both exams and their possible predictive value for prostate cancer diagnosis (Table-3). The graphic analysis through ROC curve (Receiving operating characteristics) found the following values of area under the curve for TRIMprob, mpMRI and combination TRIMprob + mpMRI: 0.706; 0.662 and 0.741, respectively. The combination TRIMprob + mpMRI showed the highest index of area under the curve when compared to the single analysis of both methods (Figure-2).

## DISCUSSION

Epidemiological data from Brazil show that 37% of all prostate cancer diagnosed correspond to advanced disease, reinforcing the importance of population screening in our country (12). Unfortunately, PSA specificity in levels >4.0ng/mL is only 60-70% and, therefore, up to 40% of PB are unnecessary (13). Still, false negative rate is also elevated (30-40%), leading to the realization of multiple biopsies with their associated morbidity (14). In that case, the search for other diagnostic methods is justified, in order to identify patients that really need biopsy and to spare those with lower diagnostic risk of PCa.

Several studies show advances of the role of mpMRI to detect PCa (15, 16). A recent meta-analysis by Hamoen et al. showed a combined sensitivity of 88% (CI 95%, 82-93) and a specificity of 45% (CI 95%, 27-65) in studies using the PI-RADS scale (17). However, mpMRI is very limited to be used in all population due to its cost (18). In that case, TRIMprob has evolved as an alternative for patients candidate to PB. As shown by our study, TRIMprob results were consistent and the method could be used as an additional diagnostic tool to screen patients candidate to PB.

Previous studies confirmed our findings. In 2005, Belloforonte et al. presented their results in 211 patients submitted to TRIMprob and posterior PB. They related sensitivity of 95.4% and specificity of 42.7%. However, the authors included patients with more advanced disease profile, with PSA values up to 38.6ng/mL (19). In our study, we used the PSA level=10ng/mL as superior limit for inclusion, since we believe that most cases with dubious indication of PB have PSA up to this value, in special in patients with no palpable disease. Two other Italian groups also described similar results (20, 21). Gokce et al. showed in 2009 in 148 patients submitted to TRIMprob, sensitivity specificity, PPV and NPV of 76%, 61.3%, 39.6% and 88.5%, respectively. They suggest the use of TRIMprob for population screening, although with some technical difficulties (22).

Our work is innovative since demonstrated in the same group of men the results of mpMRI and TRIMprob for the detection of PCa. Both methods were useful. Area under the curve of mpMRI was 0.662 while for TRIMprob was higher, 0.706. In 7 patients with PCa, mpMRI was negative and TRIMprob was positive. It was not observed a specific pattern of Gleason score in this group of patients, suggesting that TRIMprob is useful regardless the analyzed group risk. The association of both mpMRI and TRIMprob showed an area under the curve of 0.741. It is important to stress that when both methods were associated, the only case in which PB showed PCa was related to an insignificant clinical disease (single sample with only 20% of prostate adenocarcinoma Gleason 6 (3+3)). These findings reinforce the usefulness of TRIMprob in daily practice, singly or in combination to mpMRI.

Our study has some limitations. It is a retrospective study from a single center and with low number of patients. We defined patients with non-neoplasic disease those with negative biopsies, but it is known that false negative can reach 30% at first PB. However, all patients were submitted to prostate mpMRI and targeted biopsy, increasing the accuracy of PB and reducing the possibility of false negative results. Only with a longer follow-up it will be possible to determine how many of those patients will have prostate cancer in subsequent biopsies.

## CONCLUSIONS

Our study showed that TRIMprob was an efficient predictive method for the diagnosis of prostate cancer in patients submitted to PB, with results very similar to mpMRI. When associated, TRIMprob and mpMRI had a higher accuracy than when performed singly. Since it is a more available technical method, we encourage other groups to confirm our results and to reinforce the real impact on screening patients candidate to PB, reducing the number of unnecessary biopsies.

## References

[1] Siegel RL, Miller KD, Jemal A (2016). Cancer statistics, 2016. CA Cancer J Clin.

[2] Surveillance, Epidemiology, and End Results Program 2014.

[3] United Nations DoEaSA, Population Division (2009). World population prospects: the 2008 revision.

[4] Heidenreich A, Abrahamsson PA, Artibani W, Catto J, Montorsi F, Van Poppel H (2013). European Association of Urology recommendation. Eur Urol.

[5] Loeb S, Heuvel S van den, Zhu X, Bangma CH, Schröder FH, Roobol MJ (2012). Infectious complications and hospital admissions after prostate biopsy in a European randomized trial. Eur Urol.

[6] Macefield RC, Metcalfe C, Lane JA, Donovan JL, Avery KN, Blazeby JM (2010). Impact of prostate câncer testing: an evaluation of the emotional consequences of a negative biopsy result. Br J Cancer.

[7] Puech P, Potiron E, Lemaitre L, Leroy X, Haber GP, Crouzet S (2009). Dynamic contrast-enhanced-magnetic resonance imaging evaluation of intraprostatic prostate cancer: correlation with radical prostatectomy specimens. Urology.

[8] Vargas HA, Akin O, Shukla-Dave A, Zhang J, Zakian KL, Zheng J (2012). Performance characteristics of MR imaging in the evaluation of clinically low-risk prostate cancer: a prospective study. Radiology.

[9] Vedruccio C (2001). Electromagnetic analyzer of anisotropy in chemical organized systems.

[10] Bellorofonte C, Vedruccio C, Tombolini P, Ruoppolo M, Tubaro A (2005). Non-invasive detection of prostate cancer by electromagnetic interaction. Eur Urol.

[11] Quinlan MR, Casey RG, Flynn R, Grainger R, McDermott TE, Thornhill JA (2009). A review of repeat prostate biopsies and the influence of technique on câncer detection: our experience. Ir J Med Sci.

[12] FOSP (2009). Sobrevida de pacientes com câncer no estado de São Paulo: seis anos de seguimento pelo registro hospitalar de câncer 2000 a 2005.

[13] Polascik TJ, Oesterling JE, Partin AW (1999). Prostate specific antigen: a decade of discovery--what we have learned and where we are going. J Urol.

[14] Djavan B, Remzi M, Schulman CC, Marberger M, Zlotta AR (2002). Repeat prostate biopsy: who, how and when?. a review. Eur Urol.

[15] Komai Y, Numao N, Yoshida S, Matsuoka Y, Nakanishi Y, Ishii C (2013). High diagnostic ability of multiparametric magnetic resonance imaging to detect anterior prostate câncer missed by transrectal 12-core biopsy. J Urol.

[16] Thompson JE, Moses D, Shnier R, Brenner P, Delprado W, Ponsky L (2014). Multiparametric magnetic resonance imaging guided diagnostic biopsy detects significant prostate cancer and could reduce unnecessary biopsies and over detection: a prospective study. J Urol.

[17] Hamoen EH, Rooij M de, Witjes JA, Barentsz JO, Rovers MM (2015). Use of the Prostate Imaging Reporting and Data System (PI-RADS) for Prostate Cancer Detection with Multiparametric Magnetic Resonance Imaging: A Diagnostic Meta-analysis. Eur Urol.

[18] Rooij M de, Crienen S, Witjes JA, Barentsz JO, Rovers MM, Grutters JP (2014). Cost-effectiveness of magnetic resonance (MR) imaging and MR-guided targeted biopsy versus systematic transrectal ultrasound-guided biopsy in diagnosing prostate cancer: a modelling study from a health care perspective. Eur Urol.

[19] Bellorofonte C, Vedruccio C, Tombolini P, Ruoppolo M, Tubaro A (2005). Non-invasive detection of prostate cancer by electromagnetic interaction. Eur Urol.

[20] Da Pozzo L, Scattoni V, Mazzoccoli B, Rigatti P, Manferrari F, Martorana G (2007). Tissue-resonance interaction method for the noninvasive diagnosis of prostate cancer: analysis of a multicentre clinical evaluation. BJU Int.

[21] Tubaro A, De Nunzio C, Trucchi A, Stoppacciaro A, Miano L (2008). The electromagnetic detection of prostatic cancer: evaluation of diagnostic accuracy. Urology.

[22] Gokce O, Sanli O, Salmaslioglu A, Tunaci A, Ozsoy C, Ozcan F (2009). Tissue Resonance Interaction Method (TRIMprob) has the potential to be used alongside the recognized tests in the screening protocols for prostate cancer. Int J Urol.

